# Notes on Two Hundred and Twelve Consecutive Operations in Four Thousand Six Hundred and Ten Cases among the Medical Patients, Bristol Royal Infirmary

**Published:** 1901-12

**Authors:** E. H. Edwards Stack

**Affiliations:** House Physician.


### NOTES ON TWO HUNDRED AND TWELVE CONSECUTIVE OPERATIONS IN FOUR THOUSAND SIX HUNDRED AND TEN CASES AMONG THE MEDICAL PATIENTS, BRISTOL ROYAL INFIRMARY.

BY

E. H. Edwards Stack, M.B. Cantab, F.R.C.S.,
*House Physician.*

(Continued from p. 233.)

**Paracentesis Abdominis.**—It is very important in performing this operation to pay particular attention to asepsis, as peritonitis has been frequently known to follow it. It has been taught that it is best not to empty the abdomen too rapidly for fear of collapse, but this fear seems to be groundless. Southey's tubes used to be in vogue, so as to drain the fluid off slowly, but they have now fallen into disuse. Considering how often the abdomen is very rapidly emptied when opened by surgeons on the operating-table, not only for ascites, but in removing ovarian cysts, and this without any collapse, it is unlikely that the draining of fluid by even a large trocar should do harm. It used to be a very common method to put the patient in a chair during the tapping, so that any sign of faintness could be easily remedied by letting him lie down; and no doubt in this position faintness came on more easily than when lying, and this may have given rise to the groundless fear above mentioned. The middle line between the navel and the pubes is the best situation; but the old position outside the deep epigastric artery, which was commonly used, is sometimes an advantage, when in some cases the abdomen does not drain so well from the median point. It has also been customary to put a binder on, and keep this tight, to avoid the too sudden lowering of intra-abdominal pressure; but this is unnecessary, although it is often of advantage in hastening the evacuation and getting rid of the later portions of the fluid. Sometimes the abdomen, although full of fluid, is resonant all over the front; but this is no contra-indication for choosing the point of election for inserting the trocar. Unless the intestine is adherent to the abdominal wall it will not be injured, and it is just as likely to be adherent at a dull spot as at a resonant one, the dulness being proof, not of the absence of intestine, but of the fact that the intestine is collapsed. There is never any need to be in a hurry to tap an abdomen for ascites, as there is in the chest; it is done much more to relieve symptoms than with the idea of cure. Although in a few cases the fluid does not re-collect, it generally does so; but in the interval the patient is much relieved. Ascites may be due to a failing heart, and as a rule these cases receive but temporary benefit; it may occur, though not very commonly, with the general anasarca of Bright's disease, and in this case the same remark will apply. It may be due to malignant disease; and although it is usually by this time quite beyond the reach of surgery, yet it may be of very slow development, and months, or even years, may pass during which the patient has to be repeatedly tapped, with much relief of the symptoms of pressure each time, and the fluid is usually clear. The disease most benefited by abdominal paracentesis undoubtedly is chronic simple peritonitis, with or without associated cirrhosis of the liver; and in these cases, after repeated tappings, the fluid may cease to re-accumulate, or even after the first it may not return. In cirrhosis without peritonitis, it is very seldom that the patient lives long enough to require a second operation. Many of the cases in the table are labelled cirrhosis because this was the only point which could for certain be diagnosed, but it is generally recognised now that the cases requiring constant tapping are always associated with chronic peritonitis. Tubercular peritonitis with ascites is benefited by this proceeding, but for some as yet unexplained reason it seems much better to let the fluid out by a scalpel-incision than with a trocar. It is very important after tapping to close the hole as firmly as possible. This is best done by a small piece of absorbent wool, and two or three long pieces of strapping carefully and firmly applied. If leaking can be prevented during the first twenty-four hours, it will probably not take place later; but if it once starts it often goes on for a long time, and is very distressing, as the patient is continually kept wet. One might think that sepsis could more easily occur in a case like this, but I have not heard of or seen a case in which this happened. I believe in a case which looked like one that might leak it would be a good plan to put in one deep stitch of silkworm gut to ensure closure, but I have no practical experience of this.

**Incisions made in Legs for Œdema.**—This proceeding, I fancy, is not so commonly employed as it used to be. It is most generally of use in heart cases, and is often of great benefit. In several cases in which paracentesis of the abdomen had been performed, and this was done at the same time or later, it seemed to have a much more beneficial effect than letting the fluid out of the peritoneal cavity. This may be because the fluid is let out of the tissues, and so continues to relieve the circulation; while the abdominal fluid has, so to speak, lost its direct contact with the blood stream. As it continues to flow for some time, it is a more constant relief than tapping the abdomen. The best method is to make one three-inch incision into each limb on the inner side near the ankle, and keep it if possible from suppurating, as this quickly terminates the flow. It is not painful; but the constant flow into the dressings is a nuisance, and sometimes cellulitis arises. Southey's tubes sometimes act very well instead; but they often become blocked up, and consequently are not so generally useful.

**Paracentesis Pericardii.**—This has only been performed in one case. A boy admitted with a first attack of rheumatism developed an acute pericardial effusion; and while about as ill as he could well be with this, a trocar was introduced and 5½ ounces of clear serum withdrawn. He was almost instantly relieved, and for a time he improved very much; there was no return of the fluid. Three or four weeks later an acute attack of heart failure set in, from which he died. The pericardium was found almost universally adherent. It seems to be an uncommon thing for children to get a large effusion of serum in

| Volume. | Folio. | Sex. | Age. | Disease. | Amount in ounces drawn off. | Remarks. |
|---|---|---|---|---|---|---|
| 49 | 647 | F | 42 | Morbus Cordis | 240 | Much improved. Went out relieved. |
| | 730 | M | 40 | Cirrhosis Hepatis | 250 | Improved, and went out in 2 months. |
| | 797 | F | 35 | Sarcoma of Ovary | 160 | Clear fluid. Died 7 days later. |
| | 814 | M | 35 | Cirrhosis | 460 | Tapped also 4 months previously. |
| | 831 | F | 38 | Chronic Alcoholic Peritonitis | 240 | Relieved. |
| | 846 | M | 60 | ,, ,, ,, | 260 | Death from cholæmia 12 days later. |
| 50 | 880 | M | 30 | Aortic disease | 480 | Not much relieved. Death. |
| | 1045 | M | 47 | Hodgkin's disease | 160 | No relief. Died at home later. |
| 51 | 37 | F | 50 | Mitral disease | 88 | Slight relief. |
| | 76 | M | 61 | Aortic disease | 124 | Some relief. Death 2 months later. |
| | 122 | F | 47 | Cirrhosis and Peritonitis | 400 | Death 3 days later. |
| 52 | 458 | M | 47 | Carcinoma Hepatis | 416 | Much relieved. Clear. |
| | 510 | M | 48 | Carcinoma Abdominalis | 220 | |
| | | | | ,, ,, later | 322 | Not much relieved. |
| 53 | 763 | F | 60 | Supposed Ascites | 2 | Ovarian fluid. Transferred to Surgeon. |
| | 899 | F | 50 | Cirrhosis | 84 | |
| | | | | ,, later | 125 | |
| | | | | ,, later | 140 | Went out *in statu quo*. |
| | 991 | M | 14 | Ulcerative Endocarditis | 105 | No relief. Death. |
| 54 | 43 | M | 5 | Tubercular Peritonitis | 67 | |
| | | | | ,, later | 60 | Afterwards opened & drained by Surgeon. Cured. |
| | | | | ,, later | 157 | |
| | | | | ,, later | 190 | |
| | 269 | M | 53 | Cirrhosis and Peritonitis | 187 | |
| | | | | ,, ,, later | 216 | |
| | | | | ,, ,, later | 288 | Death. |
| | 511 | M | 10 | Acute Mediastinitis | 40 | Like mustard and water. |
| | | | | ,, later | 32 | Still opalescent, but clearer. Death. |
| 56 | 734 | M | 50 | Mitral disease | 181 | Not relieved. Death. |
| | 834 | F | 36 | Chronic Peritonitis | 165 | Relieved. |
| | 952 | F | 57 | Chronic Peritonitis | 258 | Relieved. |
| | 1039 | M | 52 | Cirrhosis and Peritonitis | 400 | Morison's operation. Cured. |
| 58 | 583 | M | 49 | Cirrhosis | 98 | Relieved. |
| 59 | 722 | M | 40 | Cirrhosis | 290 | Not relieved. Death 6 weeks later. |
| | 734 | F | 40 | Cirrhosis | 134 | Morison's operation. Death. |
| | 945 | M | 38 | Cirrhosis | 220 | Relieved. |
| 60 | 48 | M | 71 | Ascites | 112 | No change. |
| | 109 | F | 44 | Cirrhosis | 64 | No change. |
| 61 | 413 | F | 4 | Portal thrombosis in Liver | 65 | No relief. Death. |

acute rheumatism; in fact, pericardial effusion at any age is much rarer than it was twenty years ago, probably due to the introduction of salicylates as a mode of treatment; but this opinion is not universally held.

**Galvano-Puncture for Thoracic Aneurysm.**—This has been performed in one case, but without benefit, and no difference was noticed during the operation on two occasions. Six and eight positive needles for twenty minutes were used.

**Exploration of Joints.**—On several occasions some fluid has been drawn from a joint affected with rheumatoid arthritis. No harm resulted, and no micro-organisims were found. Two of these were very acute cases in which the knee-joint was affected, but most were cases of chronic hydrops articuli.

**Lumbar Puncture.**[1]—This has been done on about twenty occasions. None of them had any effect on the patient for bad or good; but many were of great value in diagnosis and, moreover, what is of more importance to the patient or his friends, in prognosis. The operation is very easily performed, and is, in fact, much simpler and less painful than exploring a chest. A good many cases of cerebro-spinal meningitis with mixed infection have been described, but this occurred only in two cases, one of which had tubercular meningitis as well as the presence of Weichselbaum's diplococcus. The other was a case of cerebro-spinal meningitis, the above-mentioned micro-organism being present, and in addition a positive Widal was obtained, although there were no other certain signs of enteric fever found.

**Acute Dyspnœa necessitating Operation.**—The eight cases in the table have all occurred in patients admitted medically, and operation had to be performed suddenly. They do not include any cases of diphtheria. There are three cases of retropharyngeal abscess. The first had been ill a week, and was very septic-looking. Nothing could be seen in the pharynx except mucus; the patient was too weak to talk or cry, except in a whisper. It was assumed to be diphtheria, and an incision was made in the skin, luckily without an anæsthetic, for the child immediately cried with such a good voice that laryngitis was excluded, and

[1] Six of these cases have already been reported in this *Journal*, March, 1901.

### LUMBAR PUNCTURE.

| Vol. | Folio. | Sex. | Age. | Disease. | Nature of Fluid and Micro-Organisms. | Remarks. |
|---|---|---|---|---|---|---|
| 51 | 439 | M | 16 | C. S. M. ... ... .. ... ... ... ... | Opalescent, W. | Death. |
| | 475 | M | 45 | C. S. M. ... ... ... ... ... ... ... | Clear, W. | Four previous punctures were clear and sterile. Death. |
| | 481 | F | 9 | C. S. M. ... ... ... ... ... ... .. | Clear, Sterile. | Not done till child was convalescent. Recovered. |
| | 491 | M | 7 | C. M. S. ... ... ... ... ... ... ... | Turbid, W. | Recovered. W. also in fluid from Herpes. |
| | 500 | M | 47 | Cerebral Softening ... ... ... ... | Clear, Sterile. | Death. |
| | 517 | M | 2 | Meningitis ... ... ... ... ... ... | Clear, Sterile. | Death. |
| 60 | 57 | F | 21 | C. S. M. ... ... ... ... ... ... ... | Turbid, W. | Death. |
| | 227 | M | 15 | C. S. M. ... ... ... ... ... ... ... | Turbid, W. | Cured. |
| | 254 | M | 8 mo's | C. S. M. .. ... ... ... ... ... ... | Turbid. Bacillus found. | Probably contamination. Death. |
| | 273 | F | 16 | C. S. M. ... ... ... ... ... ... ... | No Fluid obtained. | Point of needle broken. Death. |
| 61 | 631 | F | 9 | Tubercular Meningitis .. ... ... ... | Clear, Sterile. | Death. |
| 61 | 404 | F | 9 | C. S. M. and Enteric ... ... ... ... | Clear, W. | Plus Widal. Slow recovery. |
| 61 | 162 | F | 7 | C. S. M. and Tubercular Meningitis | Clear, W. | Death. No Spinal Meningitis. |
| | 760 | F | 8 | ? Cerebral Tumour ... ... ... ... | Clear, Sterile. | Improved. |
| | 773 | M | 11 | Meningitis ... ... ... ... ... ... | Clear, Sterile. | Improved. |

C. S. M. signifies Cerebro-spinal Meningitis. W. signifies that the diplococcus of Weichselbaum was found.

the abscess was then discovered and opened; but though breathing and swallowing were relieved, the child died a few days later of septic absorption. The second seemed to be broncho-pneumonia on admission, but some days later urgent dyspnœa came on, which proved to be due to an abscess similar to the last case. This was opened below the sterno-mastoid; she slowly recovered. The third was supposed to be diphtheria. The pharynx was examined first, in order to eliminate abscess, which however was discovered and treated. These three cases had all arisen from some cause which was never discovered; they were certainly not due to caries of the spine. They were all drained with ease: a small skin incision was made below the ear behind the sterno-mastoid, and then a pair of pressure-forceps pushed in till the point was felt by the finger in the pharynx on the other side of the mucous membrane, and a tube inserted. The quality of the voice when obtainable is the most useful sign for excluding laryngitis as a cause of dyspnœa.

**Bleeding.**—There have been so few cases, that it is not worth while tabulating them. A few for cerebral hemorrhage with lividity have not seemed to be of any practical value. More, for acute heart failure, have caused considerable relief, two of which certainly saved the patients' lives. Twice with acute heart failure the right ventricle was aspirated with an exploring needle; several drachms were easily removed, but without benefit. Although many have written that venesection has been allowed to fall too much into disuse, yet all attempts to revive it as a common procedure have failed, and presumably the majority who seldom practise it are right. One exception to this rule must be made in favour of eclampsia, in which it is rightly recognised as a very valuable therapeutic agent.

**Abscesses.**—Many times have incisions been made in quinsy. In more than half pus was not found, and in some of these great relief followed the puncture; in others, none; and in others, again, the symptoms seemed to be made worse. In those in which pus was found the relief was often immediate, but then these would have shortly discharged themselves. In not a few the abscess filled again, and either was punctured

### ACUTE DYSPNŒA NECESSITATING OPERATION.

| Vol. | Folio. | Sex. | Age. | Disease. | Operation. | Remarks. |
|---|---|---|---|---|---|---|
| 50 | 1006 | M | 17 | Acute Goitre ... ... ... ... ... ... | T. | Excision later. |
| | 1063 | F | 29 | Syphilitic Laryngitis and Pneumonia | T. | Died quite suddenly. Operation failed to restore breathing. |
| | 1068 | F | 9 | Tubercular Laryngitis ... ... ... ... | I. and T. | Death some weeks later. |
| 52 | 505 | M | 54 | Carcinoma of Œsophagus ... .. ... | T. | Dyspnœa came on quite suddenly from the bursting of an abscess. Lived comfortably 2 weeks. |
| 54 | 163 | M | 2 | Retropharyngeal Abscess. Admitted (?) Diphtheria | Opened behind sterno-mastoid. | Breathing quite relieved. Died of Toxæmia. |
| 56 | 912 | F | 2 | Retropharyngeal Abscess ... ... ... | Opened as last case. | Recovered. |
| | 938 | F | 4 mo's | Retropharyngeal Abscess ... ... ... | Opened as above. | Cured. |
| 59 | 893 | M | 58 | Double Abductor Palsy ... ... ... | I. and T. | No cause discovered for the disease. Improved and tube left out. Still has dyspnœa six months later. |
| 61 | 428 | M | 3 | Nutshell in Larynx ... ... ... ... | T. | Shell found immediately. |

T. signifies Tracheotomy. I. signifies Intubation.

a second time or discharged itself. Summing up all I have seen, I would say that incision seldom is contra-indicated and often is beneficial, but the routine practice of puncturing a quinsy is not of as great value as is generally supposed. I have known medical men who have often had this disease, which unfortunately has a tendency to recur, say that they were more satisfied, after trying both methods, to leave it to nature. Several parotid buboes have had to be opened, although parotitis apart from mumps most usually subsides. The cases which develop buboes, as is well known, are those with some abdominal disease, most often of a septic nature; but it is also noticeable that they seldom appear, except in patients suffering from a dry, foul mouth, which no doubt shows the ætiology to be infection through Stenson's duct. Two cases occurred of pyæmic abscesses following pneumonia; in both the pneumococcus was the only micro-organism found. Both patients recovered.

